# A systematic survey: role of deep learning-based image anomaly detection in industrial inspection contexts

**DOI:** 10.3389/frobt.2025.1554196

**Published:** 2025-06-23

**Authors:** Vinita Shukla, Amit Shukla, Surya Prakash S. K., Shraddha Shukla

**Affiliations:** ^1^ Drone Lab, Centre for Artificial Intelligence and Robotics, Indian Institute of Technology Mandi, Mandi, Himachal Pradesh, India; ^2^ Drone Lab, School of Mechanical and Materials Engineering, Indian Institute of Technology Mandi, Mandi, Himachal Pradesh, India

**Keywords:** image anomaly detection, industrial anomaly detection, deep learning, anomaly dataset analysis, surface defect detection

## Abstract

Industrial automation is rapidly evolving, encompassing tasks from initial assembly to final product quality inspection. Accurate anomaly detection is crucial for ensuring the reliability and robustness of automated systems. The intelligence of an industrial automation system is directly linked to its ability to detect and rectify abnormalities, thereby maintaining optimal performance. To advance intelligent manufacturing, sophisticated methods for high-quality process inspection are indispensable. This paper presents a systematic review of existing deep learning methodologies specifically designed for image anomaly detection in the context of industrial manufacturing. Through a comprehensive comparison, traditional techniques are evaluated against state-of-the-art advancements in deep learning-based anomaly detection methodologies, including supervised, unsupervised, and semi-supervised learning methods. Addressing inherent challenges such as real-time processing constraints and imbalanced datasets, this review offers a systematic analysis and mitigation strategies. Additionally, we explore popular anomaly detection datasets for surface defect detection and industrial anomaly detection, along with a critical examination of common evaluation metrics used in image anomaly detection. This review includes an analysis of the performance of current anomaly detection methods on various datasets, elucidating strengths and limitations across different scenarios. Moreover, we delve into the domain of drone-based, manipulator-based and AGV-based anomaly detections using deep learning techniques, highlighting the innovative applications of these methodologies. Lastly, the paper offers scholarly rigor and foresight by addressing emerging challenges and charting a course for future research opportunities, providing valuable insights to researchers in the field of deep learning-based surface defect detection and industrial image anomaly detection.

## 1 Introduction

In the complex manufacturing environment of industrial production, ensuring product quality is paramount. Technological constraints and cluttered operational environments can hinder detection of surface defects and anomalies in automation processes, potentially leading to costly product recalls and safety risks. In these complex industrial environments, detecting anomalies in surfaces of industrial components and automation processes is crucial for advancing industrial automation. Over the years, defect detection methodologies have evolved from traditional manual inspection practices to sophisticated automated systems, driven by advancements in computer vision and deep learning.

The emergence of Industry 4.0, characterized by the seamless integration of cyber-physical systems, cloud computing, and artificial intelligence, heralds a new era of intelligent manufacturing. Within this paradigm, the imperative for intelligent defect detection systems becomes increasingly pronounced, poised to revolutionize production processes, elevate product quality, and optimize resource utilization. A key part of this transformation is visual anomaly detection. This involves using powerful computer techniques to examine huge amounts of visual data for small errors or unusual patterns that human perception might miss.

Traditional and advanced defect detection methodologies (Bhandarkar et al., 2024), spanning from manual visual inspection to specialized non-destructive testing techniques, have long served as the cornerstone of quality assurance practices across diverse industrial sectors. However, the advent of machine vision systems, driven by advancements in image processing algorithms and cutting-edge technologies, has precipitated a paradigmatic shift in automated defect detection. This shift has empowered industrial processes with high precision and efficiency in detecting anomalies within visual data streams.

The development of deep learning algorithms, especially convolutional neural networks (CNNs) (O’Shea and Nash, 2015) and recurrent neural networks (RNNs) (Sherstinsky, 2018) has further catalyzed advancements in industrial automation. Cheng (2023), Radford et al. (2018), and Gao et al. (2023) used these machine vision advancements to improve defect detection capabilities, transcending previous limitations by discerning intricate patterns and anomalies with remarkable accuracy. Within the domain of anomaly detection Table 1 provides a detailed overview of various objects that can be identified as anomalies or defects in different application domains. A supervised, unsupervised and semi-supervised learning methods each offer distinct yet complementary approaches for anomaly detection, leveraging labeled and unlabeled data to varying extents to uncover deviations from expected norms.

**TABLE 1:** Object as an anomaly defect.

Reference name	Objects
MVTec AD (Bergmann et al., 2021)	Multiple materials
Solar Cells (Brabec et al., 2018)	Electroluminescence (EL) images
Solar Cell Images (Pratt et al., 2023)	Electroluminescence (EL) Images
Magnetic Tile Surface Defects (Huang et al., 2018)	Tile
Concrete Cracks on Bridge Decks (Dorafshan and Maguire, 2017)	Bridge Decks
Civil Structural Inspections (Dorafshan et al., 2016)	Bridge Decks
Bridge Crack Detection (Peng et al., 2018)	Bridge crack

This review focuses on providing a comprehensive survey of defect detection methodologies, traversing the range of supervised, unsupervised, and semi-supervised learning paradigms. By meticulously dissecting the complexity of each approach, we aim to clarify their theoretical basis, algorithmic frameworks, and practical implications in real-world industrial settings. Through a rigorous analysis and synthesis of existing literature, this paper seeks to distill key insights, identify prevailing challenges, and describe future research directions to propel the field of industrial defect detection towards new frontiers of innovation and excellence.

In the ensuing sections, we embark on a systematic exploration of defect detection methodologies, navigating through traditional machine vision techniques to cutting-edge deep learning-based approaches. We scrutinize the efficacy of supervised, unsupervised, and semi-supervised learning paradigms in anomaly detection, unraveling their intricacies and applicability across diverse industrial domains. Furthermore, we delve into the complexities of dataset curation, model evaluation metrics, and real-world challenges encountered in industrial defect detection. By furnishing a comprehensive understanding of defect detection methodologies, this review endeavors to empower researchers, practitioners, and industry stakeholders to navigate the complex terrain of industrial quality assurance with precision and confidence.

### 1.1 Research relevance

This research highlights the significance of image anomaly detection, focusing on supervised, unsupervised and semi-supervised approaches. Table 2 Lists the keywords used for paper searching. Methodologies such as density estimation, one-class classification, image reconstruction, and self-supervised classification are explored for image-level anomaly detection. In pixel-level anomaly detection, image reconstruction methods using convolutional autoencoders and deep generative models like VAEs and GANs, as well as feature modeling methods utilizing pre-trained deep convolutional features, are investigated. Recent advancements include gradient-based attention mechanisms and interpretable deep generative models, prompted by the need for improved detection algorithms highlighted by benchmark datasets like MVtec AD. These approaches contribute to enhancing anomaly detection’s efficiency and effectiveness in identifying anomalies in images. The survey provides a holistic view of the evolution of image anomaly detection techniques, from early methods to the most recent state-of-the-art approaches. This review focuses on the industrial applications of anomaly detection techniques. Overall, this review provides in-depth information on anomaly detection techniques using drones for outdoor industrial inspections and extending these techniques to indoor industrial inspections using automatic guided vehicles and manipulators. This broad perspective helps researchers understand the historical development of anomaly detection ideas in real-world applications.

**TABLE 2:** Keywords used for paper searching [*Acronym AD: Anomaly Detection*].

Type	Keywords
AD	Image Anomaly Detection
Defect types	defects, surface, crack
Application	civil structure, building, bridge, pipe
Domains	Inspection, Industries, Infrastructure
Algorithms	Deep Learning, CNN, Supervised
	Unsupervised

### 1.2 Contribution

This review provides unique contributions that set it apart from others in the field. Given the critical importance of anomaly detection across various industrial domains for inspection purposes. Our distinct contributions can be outlined as follows:

1. Synthesize prior research in the field, encompassing existing algorithms.
2. Offer a concise overview of the contributions made by previous surveys and reviews.
3. The review delves into research methodologies, covering supervised, unsupervised, and semi-supervised approaches. An extensive examination of deep learning-based methods for image anomaly detection is provided.
4. Prominent anomaly detection datasets such as surface defect detection and industrial anomaly detection are introduced.
5. The review includes an in-depth evaluation of the performance of current anomaly detection methods across diverse datasets.
6. This review explores the industrial applications of robotic inspection, including drones, AGVs, and manipulators, to detect anomalies, enhance precision, and improve adaptability in real-world settings.
7. Addressing the limitations of existing approaches, recommendations and future research directions are offered to overcome challenges in the field.

### 1.3 Research structure

**FIGURE 1:**
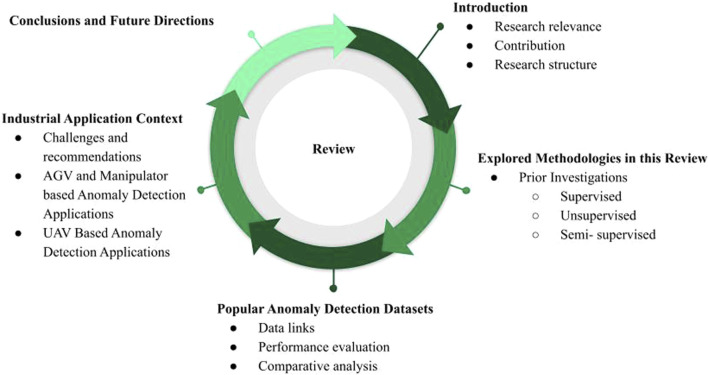
Framework of this survey.

The structure of this article unfolds as shown in Figure 1, Section 2 presents the summary of explored methodologies, while Section 3 delves into the popular anomaly detection datasets and their source and evaluation of their performance and comparative analysis of methods, followed by Industrial Application Context and outlined the challenges, recommendations in Section 4. In Section 4, we explore Unmanned Aerial Vehicle-based anomaly detection using deep learning and explored AGV(Automated Guided Vehicle) and manipulator based anomaly detection applications. Finally, Section 5 draws conclusions and future directions of existing approaches.

## 2 Explored methodologies in this review

### 2.1 Prior investigations

In the realm of anomaly detection within industrial images, we delve into the remarkable strides made, particularly excluding domains like action recognition and video anomaly detection (Yang Z. et al., 2023; Qasim and Verdu, 2023). Initially, statistical methods dominated, assessing pixel value distributions through techniques such as histogram analysis (Bansod, 2020), co-occurrence matrices (Pastor-López et al., 2019; Krishnand et al., 2022; Ishida et al., 2023), and local binary patterns. Subsequently, structural methods emerged, focusing on texture element characterization to represent defect spatial placement rules. Meanwhile, filter-dependent approaches applied filter banks and operators like sobel, canny, and gabor to compute energy responses, proving useful in cross-domain extraction but less adept with random textured images. In the era of neural networks and machine learning, supervised algorithms gained traction, including Neural Networks, Support Vector Machines (SVM) and k-Nearest Neighbors (k-NN). With a recent surge in deep learning-based approaches, data-driven models, whether through image-level classification or refined object localization, offer promise in anomaly detection. However, these models are challenged by limited training data coverage and labeling errors (Li et al., 2023).

In the domain of industrial anomaly detection, previous investigations have explored various methodologies, each with distinct focuses and approaches. Existing surveys have covered a wide range of topics, as summarized in Table 3, ranging from classical algorithms to deep learning-based methods, hardware and software devices, specific solutions for visual processing methods, and surface defect detection systems (Huang et al., 2018) for different materials. These surveys have categorized methods based on underlying principles, detection materials used, and defect detection techniques, including histogram-based, color-based, segmentation-based, frequency domain operations, texture-based detection, sparse feature-based operations, and image morphology operations. Notably, while some methods like (Carrara et al., 2021) have emphasized GAN-based algorithms and unsupervised methods, comprehensive summaries of recently emerged unsupervised approaches are lacking. To bridge this gap, this review aims to provide a systematic categorization of state-of-the-art algorithms for visual industrial anomaly detection, covering reconstruction-based, normalizing flow (NF)-based, representation-based, data augmentation-based, algorithm enhancement, transfer learning, feature engineering, and data augmentation approaches. Supervised, semi-supervised, and unsupervised deep learning algorithms have been investigated, with attention to different network architectures and methodological intersections.

**TABLE 3:** Review papers on image-based anomaly detection.

Paper title
Survey on Deep Industrial Image Anomaly Detection (Liu et al., 2024)
Survey on Deep Learning-Based Crowd Anomaly Detection (Rezaee et al., 2021)
Literature Review on Deep CNN-Based Visual Defect Detection (Jha and Babiceanu, 2023)
Review of GAN-Based Anomaly Detection (Xia et al., 2022)
Survey on Surface Defect Detection Methods for Industrial Products (Bai et al., 2024)
Review of CNN-Based Surface Defect Detection (Cumbajin et al., 2023)
Survey on Visual-Based Defect Detection for Industrial Applications (Czimmermann et al., 2020)
Surface Defect Detection in Civil Structures: A Review (Guo et al., 2024)

#### 2.1.1 Supervised based

**FIGURE 2:**
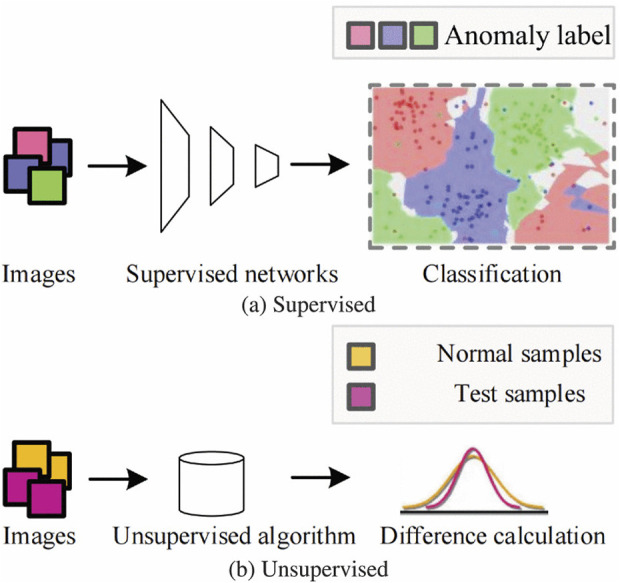
Comparison of Architectural Diagram of supervised represented in **(a)** and unsupervised algorithms represented in **(b)**.

Supervised learning constitutes the foundational approach where labeled data is utilized to train predictive models. This method involves the use of annotated datasets, where each data point is associated with a corresponding target label as shown in Figure 2.

Automated visual inspection of manufactured parts has significantly benefited from supervised learning techniques (Weiher et al., 2023), variational autoencoder based supervised technique (Kawachi et al., 2018), specifically employing Faster R-CNN methods for smart surface inspection, as referenced in studies Wang Y. et al. (2020) and Bhatt et al. (2021). These advanced techniques have also been effectively utilized in various other applications, including the automatic detection of defects in sewer pipes, as well as in the identification of cracks in masonry walls (Loverdos and Sarhosis, 2022) and defects in steel products (Ibrahim and Tapamo, 2024). By leveraging the capabilities of supervised learning, these applications demonstrate the potential for enhanced accuracy and efficiency in defect detection and quality control processes across different industries (Brasington et al., 2021).

#### 2.1.2 Unsupervised based

**FIGURE 3:**
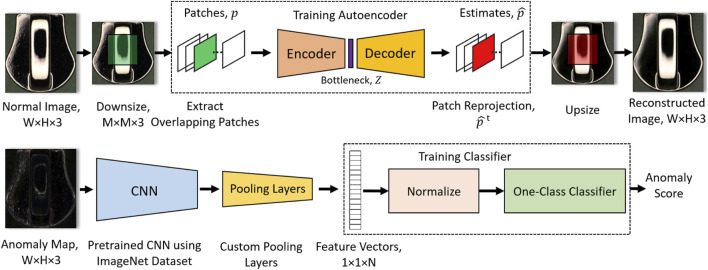
Anomaly detection autoencoder method (reprinted with permission from Saeedi and Giusti, 2023, licensed under CC-BY-NC-ND).

Unsupervised Figure 2, visual anomaly detection algorithms have garnered significant attention due to their ability to construct detection models without requiring annotated samples as shown in Lee and Kang (2022), Sun (2024), Tan and Wong (2024), and Zipfel et al. (2023).This characteristic makes them particularly well-suited for various practical applications where the collection of normal images is considerably easier and less costly compared to anomalous images. The primary advantage of these models lies in their capacity to detect a broad spectrum of anomalies by analyzing the deviations from normal samples. This enables the detection of new and unforeseen types of defects, enhancing the robustness and versatility of the detection systems (Pinon et al., 2024; Zhang F. et al., 2023; Kascenas et al., 2022). Reconstruction Based methods rely on the premise that anomalies cannot be effectively reconstructed by models trained only on normal data techniques like autoencoders (Kawachi et al., 2018; Jiang et al., 2023; Jia, 2023) as shown in Figure 3, variational autoencoders(VAEs) (Lu et al., 2024a; Lu et al., 2024b; Wang et al., 2022; Chen T. et al., 2023; Angiulli, 2023; Moon et al., 2023; Maggipinto et al., 2022; Lee and Kang, 2022; Faber et al., 2023; Zhang, 2022; González-Muñiz et al., 2022; Tao et al., 2022; Zhou et al., 2021; Ulger et al., 2021; Marimont and Tarroni, 2021; Wang X. et al., 2020) and generative adversarial networks (GANs) are commonly used (Kim, 2020; Tang et al., 2020; Han et al., 2021; Kolte, 2023; Liu R. et al., 2023; Králik et al., 2024; Ivanovska and Å truc, 2024). The reconstruction error is utilized to distinguish between normal and anomalous samples (Lin, 2023). Normalizing Flow (NF)-based methods use invertible neural networks to model the data distribution of normal samples. By transforming normal data into a simpler distribution (e.g., a standard Gaussian), anomalies can be detected based on the likelihood under the learned distribution (Hirschorn and Avidan, 2023; Gudovskiy et al., 2021).

Representation-based methods focus on learning feature representations that effectively capture the essence of normal data. Anomalies are identified by their deviation from these learned representations, methods such as deep metric learning and self-supervised learning fall under this category (Zingman et al., 2024). Data augmentation-based method used by augmenting the normal data with various transformations, these methods enhance the robustness of the detection model techniques like synthetic data generation and adversarial training are used to simulate potential anomalies and improve the model’s ability to detect real anomalies (Oyelade and Ezugwu, 2021; Motamed et al., 2021; Jain, 2022).

#### 2.1.3 Semi- supervised based

The professional process and commonly used methodologies in semi-supervised learning-based anomaly detection for images has been discussed. Semi-supervised visual anomaly detection methods leverage both labeled and unlabeled data to enhance detection performance. By using a small amount of labeled data along with a large pool of unlabeled data, these methods improve accuracy while reducing the need for extensive labeling. Key approaches include self-training, where the model iteratively labels and retrains on unlabeled data, consistency regularization, which ensures stable predictions under data perturbations, graph-based methods, which propagate labels through data similarities and generative models, which enhance detection by improving data generation processes. This hybrid approach effectively balances the benefits of supervised and unsupervised methods, making it suitable for scenarios with limited labeled data (Saheel et al., 2024; Saeedi and Giusti, 2023; Akcay et al., 2018; Rudolph et al., 2020).

An overview of the most recent techniques for detecting image anomalies is provided in Table 4 using denoising diffusion (Li et al., 2024), diffusion model (Xu H. et al., 2023; Hu and Wang, 2023) This comprehensive summary seeks to inform and advance implementation and practice within the industrial field.

**TABLE 4:** A summary of latest methodologies used for image anomaly detection.

Task	Model	Remarks
AD	Transformer (Lin et al., 2022)	The model relies on diverse pretraining and augmentation, limited coverage hampers generalization
AD	Transformaly (Cohen and Avidan, 2022)	The dual feature space approach increases memory and computation, hindering real-time deployment
AD Localization	Vision Transformers (Smith et al., 2023)	Limited data may reduce generalization and cause overfitting
Multi-class AD	Plain ViT (Zhang et al., 2024)	Struggles with subtle defects, as the model may reconstruct anomalies too well, reducing detection effectiveness
Anomaly on Textured Surfaces	ViTALnet (Tao et al., 2023)	Model performance may degrade when applied to unseen defect types or new materials
Highway vehicle AD	Attention-Based VAE (Chakraborty et al., 2023)	The model may struggle with anomalies that do not follow clear temporal dependencies
Multi-Class Industrial AD	Mixed-Attention AE (Liu and Wang, 2024)	Performance may degrade on unseen anomaly types due to reliance on learned attention patterns
Unsupervised AD	Diffusion Models (Behrendt et al., 2024)	Anomaly scoring with ensembles can introduce variability, making it hard to set optimal detection thresholds
Unsupervised Surface AD	Diffusion Probabilistic Model (Zhang et al., 2023c)	Anomaly detection relies on high-quality reconstructions, but current models often fail to achieve the necessary reconstruction fidelity
Diffusion AD	Diffusionad (Zhang et al., 2023b)	The model struggles with unforeseen or diverse anomalies
Industrial Visual AD	Dual-Attention Transformer (Yao et al., 2023)	The model’s reliance on MVTec AD and LOCO AD may limit its adaptability to diverse, unseen industrial anomalies
Unsupervised AD	A Graph-Based Model (Zhang et al., 2023a)	The integration of graph modeling with multiscale feature fitting can lead to increased computational demands
Semi-Supervised Method	MemSeg (Yang et al., 2023a)	MemSeg’s simulated anomalies may not fully represent real-world defect diversity, limiting its practical effectiveness
Image Anomaly Detection	Simplenet (Liu et al., 2023c)	SimpleNet’s reliance on pre-trained extractors may cause domain bias, leading to mismatches with target-specific data
AD	Conditioned Denoising Diffusion (Mousakhan et al., 2023)	The iterative denoising process is computationally intensive, limiting real-time use
Industrial Surface AD	Reconstruction Algorithm (Peng et al., 2024b)	Reconstruction-based methods may perfectly recreate defects due to a lack of defect samples, leading to missed anomalies
Fabric Anomaly AD	Reverse Knowledge-Distillation (ThomineS, 2024)	The dataset, though diverse, may not cover all real-world variations, limiting the model’s robustness and generalization
Surface Defect Detection	LCG-YOLO (Yu et al., 2024)	The model may face challenges in accurately identifying small-sized defects due to their subtle nature
Surface Defect Detection	Autoencoder (Getachew Shiferaw and Yao, 2024)	The two-stage training process, combined with the use of AW-SSIM and learned Perceptual Image Patch Similarity (LPIPS) losses, may introduce additional computational overhead
PCB Defect Detection	YOLO-HMC (Yuan et al., 2024)	The model becomes more complex with the incorporation of modules like HorNet, MCBAM, and CARAFE, which could raise computing requirements and have an impact on real-time processing capabilities
Industrial Defect Detection	GANomaly (Peng et al., 2024a)	Defects may be obscured by backgrounds with complex patterns, making it difficult for the model to discriminate between typical textures and abnormalities

## 3 Popular anomaly detection datasets: data links, performance evaluation, and comparative analysis

We are providing links to a diverse range of popular anomaly detection datasets, encompassing domains such as civil infrastructure with bridge crack image data and crack forest data, as well as material science with concrete crack images, tile surface defects, and steel defects. The links to popular anomaly detection datasets are provided in Table 5.

**TABLE 5:** Popular anomaly detection dataset links.

Reference	Link	Remarks
Grishin et al. (2019)	Severstal: Steel Defect Detection	• Pro: High-res steel defect images with precise segmentation. Con: Severe class imbalance
Bergmann et al. (2019)	MVTecAD	• Pro: Diverse industrial objects with multiple anomaly types. Con: Limited samples with staged defects
Tao et al. (2018)	CPLID	• Pro: Comprehensive power line insulator images with detailed annotations. Con: Inconsistent image quality
Deitsch et al. (2019)	Elpv-dataset	• Pro: Electroluminescence images of solar cells with defect annotations. Con: Limited defect diversity
Huang et al. (2018)	Tile-surface-defects	• Pro: 6 common defect types on magnetic tiles with consistent lighting. Con: Limited viewpoint variation
Dorafshan (2018)	Concrete-crackimages	• Pro: 56,000+ diverse real-world concrete structure images. Con: Binary classification lacks severity grading
Mundt et al. (2019)	Bridge crack image data	• Pro: Purpose-built for bridge crack detection with high-res images. Con: Limited geographical diversity
Shi et al. (2016)	CrackForest-dataset	• Pro: 118 images with pixel-level crack annotations. Con: Small dataset focused on pavement cracks

**FIGURE 4:**
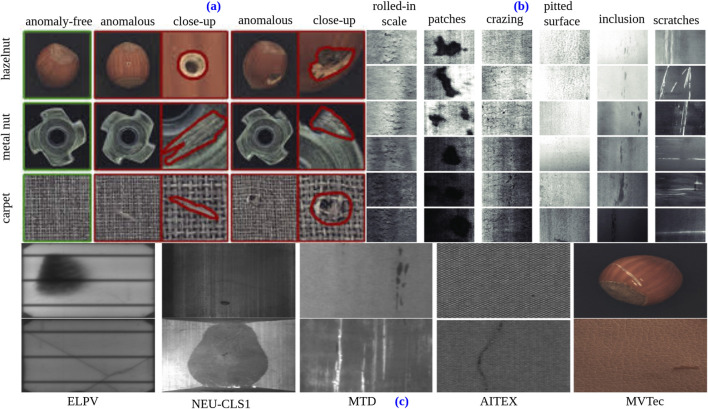
**(a)** Represents two objects (hazelnut and metal nut) and one texture (carpet) from the MVTec industrial inspection anomaly detection dataset (reproduced with permission from Bergmann et al., 2021, licensed under CC BY), **(b)** shows the examples of industrial steel surface defect (© 2018 International Federation of Automatic Control. Reproduced with the permission of IFAC from Li et al., 2018) and **(c)** shows defects of various materials in datasets (reproduced with permission from Cui et al., 2023, licensed under CC-BY-NC-ND). ELPV originally published and reproduced with permission from Deitsch et al. (2019). MTD originally published and reproduced with permission from https://github.com/abin24/Magnetic-tile-defect-datasets. (Huang et al., 2018). AITEX originally published and reproduced with permission from https://www.aitex.es/afid/ (Silvestre-Blanes et al., 2019). MVTec originally published and reproduced with permission from Bergmann et al. (2019) Copyright © 2019, IEEE.

The discussed Figure 4 provide visual examples of anomalous industrial images. (a) Illustrates examples of anomalous and anomaly-free objects (hazelnut, metal nut) and textures (carpet) from the MVTec Industrial Inspection Anomaly Detection dataset. (b) Showcases various industrial steel surface defects, including patches, crazing, pitted surfaces, scratches, and (c) illustrates the various types of defects found in different materials within the dataset. These include flaws found in photovoltaic cells, magnetic tiles, and fabric. While imperfections in photovoltaic cells may impact energy conversion efficiency, errors in magnetic tiles can jeopardize their structural integrity and magnetic qualities. Similar to this, flaws in fabric may affect its feel, longevity, or visual attractiveness. It is essential to comprehend and classify these flaws in order to maintain quality control and enhance material performances.

Algorithm Enhancement includes improvements and modifications to existing algorithms to boost their performance. Several methodologies explore innovative techniques to optimize the performance of algorithms, with a specific emphasis on improving metrics such as AUC, as illustrated in Table 6. The model enhancements might involve better loss functions, advanced training techniques, or hybrid approaches that combine multiple methods on different industrial datasets.

**TABLE 6:** Auroc average performance on different datasets.

Methods	Auroc on MVTec AD data	Dataset name
GANomaly: Semi-Supervised Anomaly Detection via Adversarial Training (Akcay, 2019)	—	MNIST, CIFAR10, X-ray security screening Data (UBA)
Natural Synthetic Anomalies for Self-Supervised Anomaly Detection and Localization (Schlüter et al., 2022)	0.972	MVTec AD
Towards Total Recall in Industrial Anomaly Detection (Roth et al., 2022a)	0.996	MVTec AD
Focus Your Distribution: Coarse-to-Fine Non-Contrastive Learning for Anomaly Detection and Localization (Zheng et al., 2022)	97.7 ± 0.4	MVTec AD, BenTech AD
Semi-orthogonal Embedding for Efficient Unsupervised Anomaly Segmentation (Kim et al., 2021)	0.982	MVTec AD, KolektorSDD, KolektorSDD2, mSTC
Transfer representation-learning for anomaly detection (Andrews et al., 2016)	—	X-ray transmission images, CASIA, MNIST
Puzzle-AE: Novelty Detection in Images through Solving Puzzles (Salehi et al., 2022)	0.776	MVTec AD
Learning and Evaluating Representations for Deep One-class Classification (Sohn et al., 2021)	0.70	MVTec AD, CIFAR10/100, Fashion MNIST, Cat-vs-Dog, CelebA
Uninformed Students: Student-Teacher Anomaly Detection with Discriminative Latent Embeddings (Bergmann et al., 2020)	0.857	MVTec AD
Towards Total Recall in Industrial Anomaly Detection (Roth et al., 2022b)	0.991 (PatchCore-25%), 0.99(PatchCore-10%), 0.99 (PatchCore-1%)	MVTec AD
Same Same But DifferNet: Semi-Supervised Defect Detection with Normalizing Flows (Rudolph et al., 2019)	0.96	MVTec AD
CSI: Novelty Detection via Contrastive Learning on Distributionally Shifted Instances (Tack et al., 2020)	—	CIFAR-10, ImageNet
Catching Both Gray and Black Swans: Open-set Supervised Anomaly Detection (Ding et al., 2022)	0.883 ± 0.008	MVTec AD
Explainable Deep Few-shot Anomaly Detection with Deviation Networks (Pang et al., 2021)	0.945 ± 0.004	MVTec AD

## 4 Industrial application context, challenges and recommendation

**FIGURE 5:**
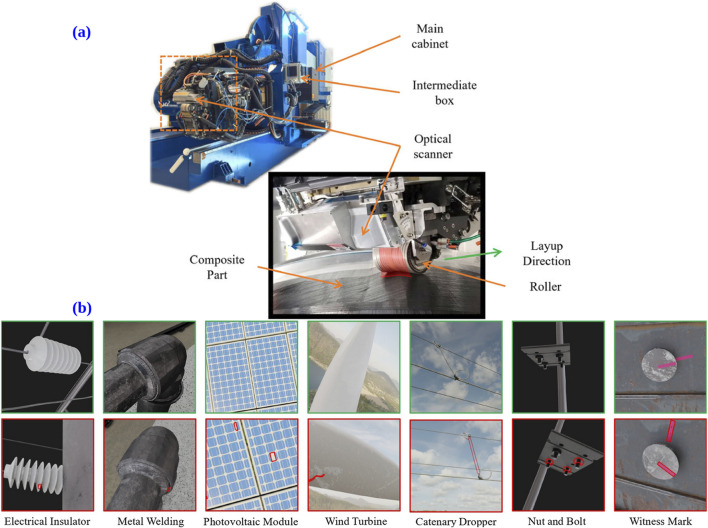
**(a)** Depicts depth images of composite carbon fiber surfaces, showcasing anomaly detection in the Automated Fiber Placement (AFP) industry (reprinted with permission from Ghamisi et al., 2024, licensed under CC BY 4.0). **(b)** Presents example images from the MIAD dataset, covering seven maintenance inspection scenarios. The test set for each scenario includes non-defective images (top row) and defective images (bottom row). The first four scenarios focus on surface anomalies, while the remaining three involve logical anomalies. Pixel-precise annotations are provided for all detected anomalies (reprinted with permission from Bao et al., 2023, Copyright © 2023 by The Institute of Electrical and Electronics Engineers, Inc.).

Anomaly detection has a wide range of significant industrial manufacturing applications (Xie et al., 2024), for instance Figure 5, highlights the detection of defects in depth images of composite carbon fiber surfaces in Automated Fiber Placement (AFP) industry. This demonstrates the potential of anomaly detection techniques to improve manufacturing efficiency and quality control in real-world maintenance and inspection scenarios. For example, identifying defects in metal welding for oil pipelines, assessing photovoltaic modules in solar power plants, and inspecting wind turbine blades in wind farms. Furthermore, they play a critical role in monitoring components like catenary droppers in overhead catenary systems, electrical insulators, nuts, bolts, and witness marks used in power transmission lines and catenary support devices.

**FIGURE 6:**
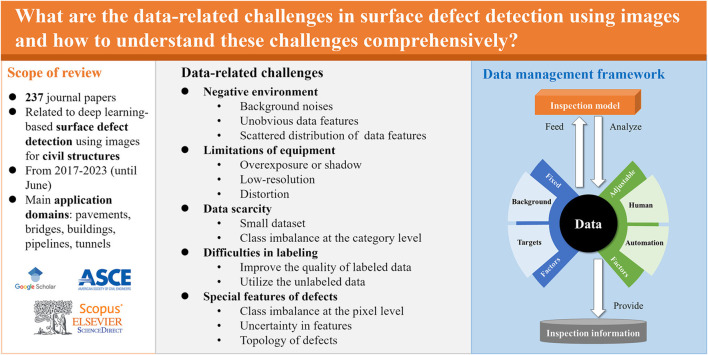
Data level challenge in surface defect detection (reprinted from Guo et al., 2024, Copyright 2023, with permission from Elsevier).

Image-based anomaly detection faces several challenges that need to be addressed for effective and efficient implementation. These challenges include real-time processing, handling small sample sizes and texture differences, detecting small targets, and managing unbalanced sample identification. Figure 6 highlights the data-level challenges in surface defect detection below, we explore these issues and provide recommendations for each.

### 4.1 Real-time problem challenge

Real-time anomaly detection requires rapid processing and analysis of image data, which can be computationally intensive and demanding.

#### 4.1.1 Recommendation

Han and Yan (2023) introduced Collaborative Representation Distance (CRD), an approach designed for practical anomaly detection, demonstrating the importance of optimized computational frameworks to achieve real-time performance.

In addition to hardware acceleration, implementing lightweight models like MobileNet can substantially improve inference speed while maintaining detection accuracy. Feng and Wang (2024) proposed an efficient object tracking algorithm based on lightweight Siamese networks, which highlights the benefits of compact architectures in real-time applications.

Further performance gains can be achieved through pruning and quantization techniques, which reduce model size and computational requirements without significantly degrading accuracy. Liang et al. (2021) provided a comprehensive survey on pruning and quantization for deep neural network acceleration, demonstrating how these techniques enhance model efficiency, particularly in resource-constrained environments.

By integrating efficient network architectures, hardware acceleration, and model compression techniques, real-time anomaly detection systems can achieve faster inference while maintaining high accuracy. These optimizations make real-time anomaly detection feasible for applications in industrial inspection, autonomous systems, and surveillance, where rapid decision-making is crucial.

To address this, optimizing the network structure for efficiency and deploying hardware acceleration techniques such as GPUs or TPUs can significantly improve processing speed (Han and Yan, 2023). Implementing lightweight models like MobileNet or using pruning and quantization techniques can also enhance real-time performance (Liang et al., 2021).

### 4.2 Small sample problem and texture difference challenge

Limited sample sizes and texture variations pose significant hurdles in accurately detecting anomalies, as models might not generalize well from sparse data.

#### 4.2.1 Combination of data distribution learning and data augmentation recommendation

Leveraging advanced data distribution learning methods and data augmentation techniques can mitigate these issues. By augmenting the existing dataset with synthetic variations (e.g., rotations, flips, color adjustments), the model can be exposed to a broader range of scenarios, enhancing its robustness. Studies have shown that combining these approaches can significantly improve detection accuracy (Fullington et al., 2024; Xu M. et al., 2023).

#### 4.2.2 Transfer learning recommendation

Transfer learning involves using pre-trained models on related tasks and fine-tuning them on the target anomaly detection task. This approach can overcome small sample problems by utilizing the knowledge embedded in models trained on large datasets, thus improving performance on smaller, specific datasets.

Limited sample sizes and texture variations pose significant hurdles in accurately detecting anomalies, as models might struggle to generalize from sparse data. When trained on small datasets, deep learning models often suffer from overfitting, leading to poor performance in real-world applications.

To address this challenge, transfer learning has emerged as a powerful technique. By leveraging knowledge embedded in models pre-trained on large-scale datasets, transfer learning enables effective feature extraction, improving performance on smaller, domain-specific datasets (Chen et al., 2023b). demonstrated its effectiveness in multimode process monitoring and anomaly detection for steam turbines, showing that adaptive transfer learning enhances model generalization across different operating conditions.

Similarly, Liu et al. (2023d) introduced SimpleNet, an efficient network designed for image anomaly detection and localization, utilizing transfer learning to mitigate the impact of limited training samples. Maray et al. (2023) further validated this approach in healthcare applications, showing that transfer learning significantly improves fall detection accuracy even with small datasets.

Iman et al. (2023) provided a comprehensive review of deep transfer learning, highlighting recent advancements and techniques that enhance model adaptability to new domains. Zhao et al. (2024) extended this concept to industrial applications, employing contrastive and transfer learning-based methods for small component inspection in assembly lines, demonstrating that prior knowledge can aid in detecting fine-grained defects.

In manufacturing, Shin (2024) introduced a material-adaptive anomaly detection framework, where property-concatenated transfer learning proved beneficial in wire arc additive manufacturing. Similarly, Cheng et al. (2022) applied transfer learning to defect detection in fabrics using a specialized U-Net architecture, improving segmentation accuracy with limited annotated data.

Medical applications have also benefited significantly from transfer learning. Lanjewar et al. (2024) fused transfer learning models with LSTMs to enhance breast cancer detection using ultrasound images, while Ani et al. (2024) explored multi-class classification of breast cancer abnormalities, proving the effectiveness of transfer learning in medical imaging scenarios.

By leveraging pre-trained models and adapting them to specific anomaly detection tasks, transfer learning provides a robust solution to the challenges posed by limited sample sizes and texture variations. It enables models to generalize better, reducing the need for extensive labeled data while maintaining high detection accuracy.

#### 4.2.3 Optimize network structure recommendation

Optimizing the network architecture to suit the specific characteristics of the anomaly detection task is crucial for improving performance. A well-structured model can better capture the nuances of small sample data and texture variations, ensuring robust detection across different scenarios.

One approach to achieving this is Neural Architecture Search (NAS), which automates the design of optimal network structures by exploring various architectures to find the most efficient model for a given anomaly detection task. Alternatively, manually designing lightweight yet effective models can also lead to significant improvements by tailoring architectures to specific dataset constraints.

Cao Y. et al. (2023) introduced a Collaborative Discrepancy Optimization framework for reliable image anomaly localization, demonstrating that optimizing network structures can enhance detection accuracy and robustness. Their approach highlights the importance of fine-tuning architectures to maximize sensitivity to abnormal patterns while maintaining computational efficiency.

Wan et al. (2024) further advanced this idea by integrating Singular Spectrum Analysis (SSA) with an optimized ResNet50-BiGRU model for image anomaly detection and prediction. Their work shows that fusing convolutional networks (ResNet50) with recurrent architectures (BiGRU) can effectively capture spatial and sequential anomaly patterns, making the detection process more precise and adaptive.

By leveraging structured network optimization techniques, either through NAS or manual model refinement, anomaly detection systems can achieve higher accuracy while remaining lightweight. This is particularly beneficial for real-time and resource-constrained applications, where both computational efficiency and detection reliability are essential.

### 4.3 Small target detection problem challenge

Detecting small anomalies or targets within images is challenging due to their minimal pixel footprint, making them difficult to distinguish from noise. Detecting small targets in images presents significant challenges due to low resolution, background noise, and scale variations. Small objects often lack distinguishable features, making it difficult for deep learning models to differentiate them from their surroundings.

#### 4.3.1 Recommendation

To address this, enhancing image resolution, leveraging multi-scale feature extraction techniques, and implementing attention mechanisms can significantly improve detection accuracy.

Multi-scale feature extraction allows models to capture fine details across different resolutions. Feature Pyramid Networks (FPN) and YOLO (You Only Look Once) are widely used architectures that enhance small target detection by leveraging hierarchical feature maps. Additionally, attention mechanisms improve focus on relevant features while suppressing background noise, thereby enhancing detection reliability. Chen et al. (2021) introduced MAMA-Net (Multi-Scale Attention Memory Autoencoder Network) for anomaly detection, demonstrating that combining attention with multi-scale memory networks enhances small target recognition in medical imaging. Their approach highlights the benefits of multi-scale learning for capturing subtle anomalies.

Xiang et al. (2023) extended this concept by proposing a Multi-Scale Attention and Dilation Network for small defect detection. By integrating dilated convolutions with attention mechanisms, their model effectively extracts fine-grained features, improving defect localization on industrial surfaces.

Xiao et al. (2022) developed a feature fusion-enhanced multiscale CNN with an attention mechanism for spot-welding surface appearance recognition. Their work demonstrated that fusing multi-resolution features allows the network to better differentiate between normal and defective welds.

Yang et al. (2020) further improved unsupervised anomaly localization by incorporating multi-scale memory modules into autoencoders, enhancing the model’s ability to detect small deviations in structured environments.

More recently, Tao et al. (2024) introduced a method for learning multi-resolution features for unsupervised anomaly localization on industrial textured surfaces. Their approach leverages multi-scale representations to detect subtle texture differences, significantly improving performance in real-world manufacturing applications.

By integrating multi-scale feature extraction, attention mechanisms, and resolution-enhancing techniques, small target detection models can achieve higher precision, making them applicable to areas such as medical imaging, industrial inspection, and remote sensing. These advancements ensure that even the smallest anomalies or defects are accurately identified, improving overall system reliability.

### 4.4 Data level challenges

Imbalanced datasets, where normal samples significantly outnumber anomalous ones, can bias the model towards the majority class, reducing the effectiveness of anomaly detection. A variety of datasets have been used to explore the challenges inherent in Industrial Anomaly Detection.

#### 4.4.1 Recommendation

Table 7 Provides a detailed overview of these datasets and their specific characteristics. Addressing this issue at the data level can involve techniques such as oversampling the minority class (anomalies) or undersampling the majority class (normal samples). Additionally, synthetic data generation methods, such as using GANs (Generative Adversarial Networks) to create realistic anomalous samples, can help balance the dataset. Implementing these strategies ensures that the model receives sufficient training on anomalies, improving its detection capabilities.

**TABLE 7:** Challenges in AD: Different datasets illustrate the challenges in the IAD field *(Acronym Y: Yes)*.

Challenge	BTAD	ELPV	Aitex	MTD-surface	KolektorSDD	DAGM	MVTecAD
Small Anomalous Data	Y	Y	Y	Y	Y		Y
Tiny Defects	Y	Y		Y	Y		Y
Appearance Inconsistency	Y	Y	Y	Y	Y	Y	Y
Textural Divergence	Y	Y	Y	Y		Y	Y

Zeiser et al. (2023) provided a detailed evaluation of deep unsupervised anomaly detection methods, emphasizing a data-centric approach for online inspection. Their work highlights how dataset characteristics significantly impact model performance, reinforcing the need for effective data-balancing techniques.

Yang Y. et al. (2023) proposed a deep learning-based anomaly detection approach that extracts representative latent features to handle cases where anomalies are low-discriminative or insufficient in quantity. Their method improves anomaly detection by enhancing feature extraction from limited abnormal data.

Mun et al. (2024) tackled data imbalance in AI-driven Clinical Decision Support Systems (AI-CDSS) by introducing U-AnoGAN, a GAN-based anomaly detection framework. Their study demonstrated that synthetically generated anomalies can significantly enhance model robustness, especially in medical applications where real abnormal samples are scarce.

Liu D. et al. (2023) introduced Deep Attention SMOTE, a data augmentation method that uses a learnable interpolation factor to generate synthetic samples for imbalanced anomaly detection in gas turbines. This approach improves detection capabilities by creating diverse training samples that prevent model bias toward the majority class.

Beyond simple resampling techniques, Cao T. et al. (2023) explored anomaly detection under distribution shift, addressing the challenge where real-world data distributions differ from those seen during training. Their findings suggest that models trained on balanced datasets must also be adaptable to dynamic, evolving data distributions to maintain high anomaly detection accuracy.

By integrating oversampling, undersampling, synthetic data generation, and distribution-aware anomaly detection methods, data-level challenges in anomaly detection can be effectively mitigated. These techniques enhance model robustness, improve generalization on rare anomalies, and ensure better real-world applicability across diverse domains, including industrial inspection, medical diagnostics, and predictive maintenance.

### 4.5 UAV based anomaly detection applications

**FIGURE 7:**
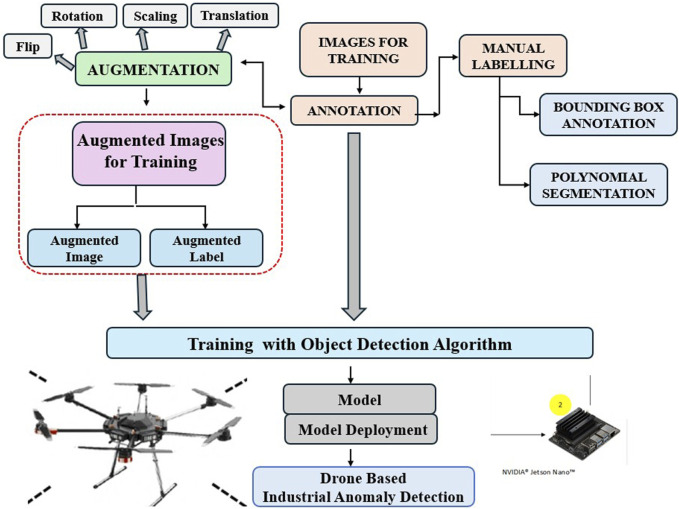
Drone based anomaly detection pipeline.

**FIGURE 8:**
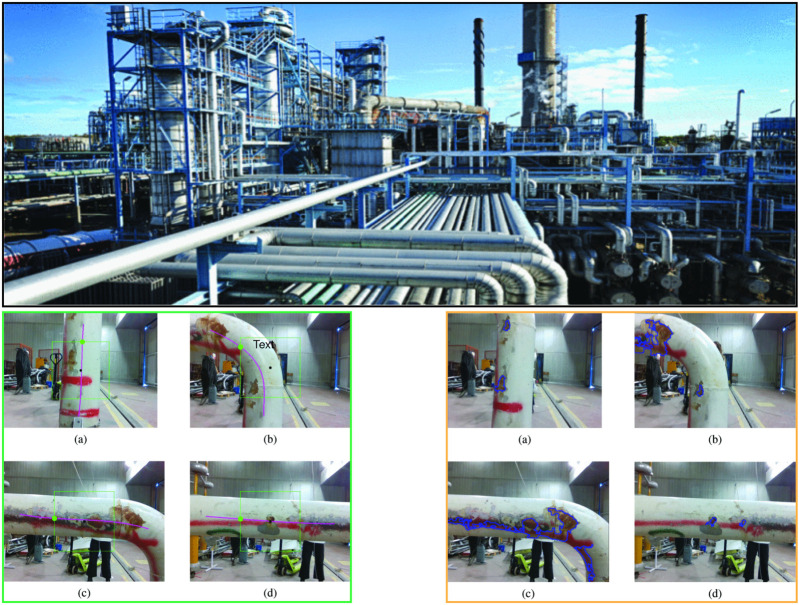
Industrial pipeline anomaly detection (reprinted with permission from Roos-Hoefgeest et al., 2023 Copyright © 2023, IEEE). (**a–d**, green border) Pipeline tracking: pink line marks the central axis, green guides the drone. (**a–d**, orange border) Corrosion detection on RGB pipeline images, defects in blue.

Unmanned Aerial Vehicle (UAV)-based anomaly detection is advancing real-time monitoring across various fields by leveraging deep learning as shown in Figure 7, a drone-based anomaly detection pipeline. Unmanned Sensing Vehicles (USVs), including UAVs, are used in environmental monitoring (Gupta, 2022; Pandya et al., 2024; Roos-Hoefgeest et al., 2023). A vision-based approach for UAVs is proposed for tracking and inspecting industrial pipelines. This system focuses on oil and gas refineries, where long pipelines at high altitudes pose challenges to human safety and operational costs. The UAV autonomously navigates the pipeline’s centerline using a depth sensor to generate control data and detect defects. Simulated and real experiments in GPS-denied environments validate the system’s effectiveness. Figure. 8 highlights anomalies detected during pipeline inspections in industrial contexts. Demonstrated inspection of vertical and horizontal structures for structural health monitoring and defect detection using UAV in contact and non-contact methods.

Precision agriculture is one main field, apart from industries, where image-based anomaly detection can be extended to simplify complex traditional methods (Subramanian et al., 2021; Mendoza-Bernal et al., 2024). Used UAVs equipped with IR camera for collecting thermal imagery from agriculture fields. Further analyzed leaf health and field water distributions from recognizing pattern from thermal imagery and CNNs. A novel adaptive sampling strategy for USVs uses spatio-temporal sequential tensor decomposition to optimize deployment for effective change detection.

For photovoltaic (PV) plant maintenance, UAVs with thermal imagers detect module defects using infrared (IR) and RGB images. The system employs SIFT for feature detection and CNNs for defect classification, achieving high accuracy and supporting real-time detection, significantly aiding PV plant maintenance (Jeffrey Kuo et al., 2023).

In traffic surveillance, UAVs address the challenges of rare events and complex backgrounds. A transformer-based future frame prediction network detects anomalies in drone videography by capturing spatial and temporal representations, demonstrating superior performance on datasets like UIT-ADrone and Drone-Anomaly (Tran et al., 2024).

**FIGURE 9:**
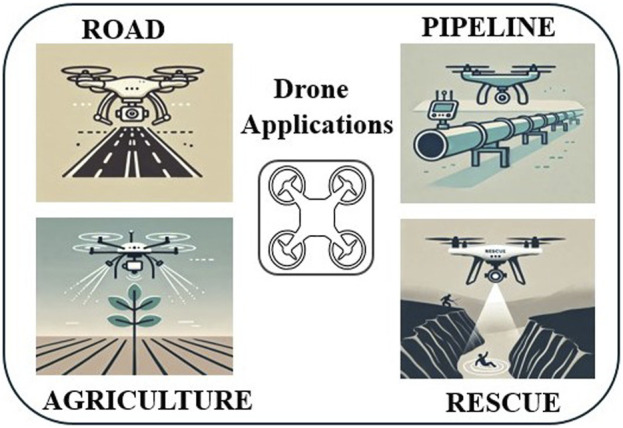
Future-Forward industrial applications.

These developments highlight the effectiveness of UAV-based anomaly detection systems, leveraging deep learning to enhance real-time monitoring and operational efficiency across various applications. Figure 9 explores emerging trends and opportunities in industrial applications, highlighting potential future developments. While UAVs are the best platform for outdoor and high-roof industrial inspections, AGVs (Autonomous Ground Vehicles) and robotic arms (manipulators) are best suited for indoor and assembly line inspections.

### 4.6 AGV and manipulator based anomaly detection applications

AGVs and robotic arms are well-suited for inspection tasks in cluttered factory and assembly line environments due to their mobility (Pérez et al., 2016). Highlights the significance of 3D vision systems in enhancing the capabilities of robotic arms for tasks such as navigation, object detection, and precise positioning. By carefully selecting appropriate vision techniques, like laser range finders or stereo imaging, based on specific task requirements and factory environments, robotic arms can be better adapted to real-world industrial settings. This enables collaborative tasks and real-time decision-making, ultimately improving overall operational efficiency. Manipulator mounted with high resolution multi-view camera can collect the images of industrial components from different views to inspect them (Galdelli et al., 2019). Khan et al. (2021) presented an automated vision-based ultrasonic Non-Destructive Testing (NDT) inspection system for manufacturing industries. To improve inspection efficiency, a practical method for accurate 3D reconstruction is proposed. Structure from Motion (SfM) techniques are utilized to generate precise 3D models of objects of interest with sub-millimeter accuracy. The paraboloid spiral tool path by robot arm has demonstrated the best accuracy of 0.43 mm (Ali et al., 2018; Surya Prakash et al., 2024a; Surya Prakash et al., 2024b). The factory setup comprises industrial components and a globally fixed camera to observe objects. The proposed methodology employs a hybrid approach that integrates classical vision techniques with deep learning algorithms. This hybrid approach enables the detection and size estimation of industrial components, facilitating subsequent actions by a 6-DOF manipulator. Inspection of objects in hazardous environments, such as high-temperature or toxic gas areas, necessitates the use of robotic manipulators controlled remotely through teleoperation. This approach provides visual and haptic feedback to operators, enabling them to safely perform intricate tasks in these challenging conditions (Naceri et al., 2019). For spacious environments, such as warehouses or large manufacturing facilities, Autonomous Guided Vehicles (AGVs) equipped with sensors are an ideal choice. Their mobility allows them to navigate complex and cluttered factory layouts efficiently. Sanchez-Cubillo et al. (2024) investigated the application of deep learning techniques to enhance the performance of Autonomous Mobile Robots (AMRs) and Autonomous Guided Vehicles (AGVs) in wide-area inspections, such as railway track and wagon loading/unloading inspections. Bao et al. (2022) presents a computer vision-based mobile robot system for inspection and maintenance of industrial pipe work, mainly focusing on colorless objects like water which are difficult to detect in cluttered environments. System leverages the reflective properties of lower temperature effusion relative to their surroundings, using dual source imaging and contour feature algorithm.

Further combinations of AGVs and robotic arms can be explored for inspection and taking appropriate actions to resolve anomalies or tag them by highlighting the defective area. Researchers can focus their efforts on factory inspections.

## 5 Conclusion and future directions

This review paper discusses the use of deep learning techniques for detecting image anomalies. It compares traditional methods with advanced deep learning approaches, including supervised, unsupervised, and semi-supervised paradigms, and addresses challenges such as real-time processing and sample imbalance, providing strategies for mitigation.

The paper surveys existing algorithms, examines different learning paradigms, and analyzes various anomaly detection methods. It highlights popular datasets, evaluation metrics, and evaluates current methods across diverse datasets, with a focus on deep learning applications in UAV,AGV,robotic manipulator-based anomaly detection. Additionally, it suggests future directions for overcoming current challenges.

Key industrial applications include few-shot anomaly detection to reduce data collection costs, enhancing robustness against labeling errors, utilizing spatial information for 3D anomaly detection, and improving model performance through synthetic data generation.

The review introduces several anomalies in different data applications like MVTec AD, Severstal Steel Defect, and Magnetic Tile Surface Defects, yarn-dyed fabric defect detection and discusses challenges such as real-time processing, small sample sizes, texture differences, small target detection, data limitation, and unbalanced sample identification. Proposed solutions include optimizing network structures, data augmentation, transfer learning, enhancing image resolution, and synthetic data generation.

In summary, this review systematically explores defect detection methodologies, aiming to help researchers and industry stakeholders enhance quality assurance in manufacturing through advanced image anomaly detection techniques.

### 5.1 Anomaly detection based future project directions

1. Crack Detection in Railway Tracks - Using drone-based image processing to identify cracks and fractures in railway tracks to help prevent accidents.
2. Surface Defect Detection in Manufacturing - Automatically detecting scratches, dents, and misalignments in industrial components to ensure quality control.
3. Underwater Crack Detection in Dams and Pipelines - Monitoring the structural integrity of underwater infrastructure using AI-powered autonomous underwater vehicles (AUVs).
4. Anomaly Detection in PCB (Printed Circuit Board) Inspection - Identifying missing components, soldering defects, and misaligned circuits in printed circuit boards.
5. Predictive Maintenance in Industrial Machinery Using Thermal/Visual Imaging - Detecting wear and tear in rotating machinery through deep learning analysis of infrared and RGB images.
6. Surface Corrosion and Rust Detection on Metal Structures - Identifying early signs of corrosion and rust on bridges, pipelines, and marine structures.
7. Food Quality Inspection in Factories - Using AI-based visual inspection to detect contamination, bruises, and deformities in food products.
8. X-ray and CT Image Anomaly Detection for Cargo and Security - Identifying concealed weapons, drugs, and smuggled items in security scans using advanced imaging techniques.
9. Optical Inspection for Textiles and Fabric Defect Detection - Detecting weaving defects, misprints, and other irregularities in textile manufacturing.
10. AI-Powered Glass Surface Defect Detection - Identifying scratches, cracks, and contamination on glass surfaces used in construction and electronics.
11. Leakage Detection in Oil and Gas Pipelines - Using thermal and hyperspectral imaging to detect small leaks in oil and gas pipelines.
12. Automated Inspection of Weld Defects - Identifying cracks, porosity, and misalignment in welds using deep learning algorithms.
13. Battery Cell Anomaly Detection in EV Manufacturing - Detecting defects in lithium-ion battery cells using thermal imaging technology.
14. Structural Health Monitoring of Bridges and Highways - Using UAV-based vision systems to identify structural anomalies and cracks in large-scale infrastructure like bridges and highways.
15. Automated Defect Inspection in Aerospace Components - Detecting defects in aerospace components to ensure safety and reliability in the aviation industry

Further research is needed on foundational models, which, as pre-trained models, show great potential in anomaly detection due to their ability to capture broad patterns and quickly adapt to new domains with high-quality representation. Their application in industrial anomaly detection is promising but still requires further exploration.
